# Response to the comment on “Association between frailty recovery and dietary variety among community-dwelling older Japanese adults: A longitudinal study from 2023 to 2024”

**DOI:** 10.1016/j.jnha.2026.100852

**Published:** 2026-04-20

**Authors:** Tamaki Hirose, Yohei Sawaya, Tomohiko Urano

**Affiliations:** a Department of Physical Therapy, School of Health Sciences, International University of Health and Welfare, Otawara, Tochigi, Japan; b Department of Geriatric Medicine, School of Medicine, International University of Health and Welfare, Narita, Chiba, Japan

Thank you for the comment by Yildirim Borazan et al. regarding our study (Article 100783) [1]. We sincerely appreciate your interest in our research and for providing important perspectives regarding the association between dietary variety and frailty recovery. We have addressed the issues raised as follows.

As you noted, the higher dietary variety score (DVS) observed in this study may reflect greater intake of plant-based foods, particularly soybean products. In fact, 64.1% of participants in the robust/improvement group reported consuming soybean products almost daily, and among the ten DVS food categories, soybean products were the only item significantly associated with frailty status in the multivariable analysis. These findings suggest that regular consumption of soybean products may be associated with frailty status [1]. Furthermore, soybean foods contain polyunsaturated fatty acids (PUFAs) and bioactive compounds that may contribute to anti-inflammatory effects and the maintenance of skeletal muscle function, providing a biologically plausible explanation for frailty recovery. In addition, reduced intake of plant-based proteins and PUFAs has been reported to be independently associated with frailty, suggesting that the qualitative aspects of diet may influence frailty [2]. PUFAs may affect skeletal muscle function through anti-inflammatory effects and anabolic actions on muscle protein metabolism. Associations between erythrocyte n-3 PUFA levels and frailty as well as subsequent onset of frailty have also been reported [[2], [3], [4], [5]]. Taken together, the higher frequency of soybean product consumption observed in this study may not simply reflect dietary variety but may also indicate underlying biological mechanisms mediated by nutrients such as PUFAs and plant-based proteins.

Furthermore, the overall quality of dietary patterns is an important factor associated with frailty. Adherence to the Mediterranean diet has been reported to be associated with a reduced risk of frailty [6,7]. In addition, longitudinal studies examining the relationship between dietary quality indices and incident frailty have shown that higher dietary quality is associated with a lower risk of developing frailty [8]. Moreover, studies involving older Japanese adults have reported associations between sarcopenia and traditional Japanese dietary patterns characterized by greater intake of soybean products, seafood, and vegetables, demonstrating that higher Japanese diet scores are associated with a lower prevalence of sarcopenia [9]. Taken together, the finding that soybean products were uniquely identified within the dietary variety assessed in this study suggests that the observed association may reflect not only the number of food items consumed, but also dietary patterns characteristic of the Japanese diet and the associated intake of nutrients such as PUFAs and plant-based proteins, which may contribute to frailty recovery through the maintenance of muscle function.

In summary, the findings of this study highlight the importance of dietary variety while also suggesting that the underlying nutritional composition and qualitative features of dietary patterns may contribute to frailty recovery. Future intervention studies are warranted to clarify whether simple and practical dietary behaviors, such as “small food add-ons” of soybean products, as suggested by the present findings, may influence frailty outcomes.

## Declaration of Generative AI and AI-assisted technologies in the writing process

The authors used AI-assisted technologies only for spelling and grammatical corrections in limited portions of the manuscript. All content was subsequently reviewed, edited, and approved by the authors to ensure accuracy.

## Funding

This study was funded by JSPS Grants-in-Aid for Scientific Research (25K14197) and the JGS Grant for Geriatric Nutrition Research supported by Otsuka Pharmaceutical Factory, Inc.

## CRediT authorship contribution statement

**Tamaki Hirose:** Writing – original draft, Writing – review & editing. **Yohei Sawaya:** Writing – original draft, Writing – review & editing. **Tomohiko Urano:** Supervision, Writing – review & editing.

## Declaration of competing interest

The authors declare that they have no conflict of interests.
